# Quality of Life in Multiple Sclerosis

**DOI:** 10.15844/pedneurbriefs-34-14

**Published:** 2020-12-04

**Authors:** Mitchell T. Williams, Lalitha Sivaswamy

**Affiliations:** 1 Departments of Pediatrics and Neurology, Children's Hospital of Michigan, Wayne State University School of Medicine, Detroit, MI

**Keywords:** Pediatric, Multiple Sclerosis, Quality of Life

## Abstract

Investigators from Karolinska Institute in Stockholm, Sweden report on their findings comparing quality of life (QoL) measures in both pediatric-onset multiple sclerosis (POMS) and adult-onset multiple sclerosis (AOM).

Investigators from Karolinska Institute in Stockholm, Sweden report on their findings comparing quality of life (QoL) measures in both pediatric-onset multiple sclerosis (POMS) and adult-onset multiple sclerosis (AOM). Data was collected from the nationwide Swedish multiple sclerosis (MS) registry between 2010 & 2019 (354 POMS; 4,740 AOM). Analyses of their findings were interpreted as showing no significant difference between POMS and AOMS regarding QoL measures in adulthood. The most significant determinants that negatively influenced QoL were relapses, severe neurologic disability, and higher MSIS-29 psychological score. Those with higher information processing efficiency and exposure to first-line DMTs were associated with higher QoL scores. The authors suggest focusing on reducing neurological disability and psychological status as potential measures to improve QoL in both POMS and AOMS. [1]

COMMENTARY. The etiology of MS is not well understood; however, diagnostic criteria and therapeutic agents have continued to advance, and further refinement has progressed over the last several decades, especially in the field of POMS. Despite such advances, there remains a great deal of morbidity in both POMS and AOMS. The physical and cognitive effects of MS have been long recognized and studied in AOMS. However, until more recently, the factors affecting QoL in POMS had primarily been under-recognized. Pediatric patients typically have a comparatively quicker recovery from relapses and longer course until the progression to permanent neurological disability does so at a younger age [2]. The lasting cognitive effects in POMS have been found to occur early on in the course of the disease, with physical disability occurring later, which contrasts to adults where both physical and cognitive effects often parallel each other in the course of the disease. This clinical course difference may initially feel counter-intuitive, as it is generally known that the pediatric brain possesses more plasticity and cognitive reserve than its adult counterpart. More recent research indicates that the onset of MS in critical neurodevelopmental periods likely has a more significant impact on cognitive function than in the adult brain, significantly earlier in the disease course [3]. Although not fixed in its makeup, the adult nervous system has completed the myelination process and most of its synaptic formation and other major critical structural pathways that may account for differences observed.

This study's impactful aspects are the large number of children they included in their cohort and the unique manner in which the QoL measures of the same disease were compared between children and adult-onset cases. Most studies involving QoL measures in children with neurological disorders compare the study cohort, which is often small, with children who do not have the disease process [4,5]. While this information is helpful, it is not as pertinent to a clinician as studying the same disease state across the life span or with onset at different ages. The authors' key finding that children with POMS experience significant impairments in several critical QoL measures is essential for practicing child neurologists. It should encourage them to adopt a multifaceted approach focusing on both psychological function and physical disability for better long-term outcomes.

## Disclosures

The authors have declared that no competing interests exist.
